# MARCO Inhibits Porcine Reproductive and Respiratory Syndrome Virus Infection through Intensifying Viral GP5-Induced Apoptosis

**DOI:** 10.1128/spectrum.04753-22

**Published:** 2023-04-20

**Authors:** Xiaoxiao Zhang, Yongjie Chen, Songbei Li, Jinling Wang, Zhan He, Jiecong Yan, Xiaohong Liu, Chunhe Guo

**Affiliations:** a State Key Laboratory for Animal Disease Control and Prevention, Key Laboratory of Zoonosis Prevention and Control of Guangdong Province, College of Veterinary Medicine, South China Agricultural University, Guangzhou, Guangdong, People’s Republic of China; b Guangdong Laboratory for Lingnan Modern Agriculture, Guangzhou, Guangdong, People’s Republic of China; c State Key Laboratory of Biocontrol, School of Life Sciences, Sun Yat-sen University, Guangzhou, Guangdong, People’s Republic of China; Changchun Veterinary Research Institute

**Keywords:** MARCO, yeast two-hybrid screening, apoptosis, glycoprotein 5, porcine reproductive and respiratory syndrome virus

## Abstract

Studying viral glycoprotein-host membrane protein interactions contributes to the discovery of novel cell receptors or entry facilitators for viruses. Glycoprotein 5 (GP5), which is a major envelope protein of porcine reproductive and respiratory syndrome virus (PRRSV) virions, is a key target for the control of the virus. Here, the macrophage receptor with collagenous structure (MARCO), which is a member of the scavenger receptor family, was identified as one of the host interactors of GP5 through a DUALmembrane yeast two-hybrid screening. MARCO was specifically expressed on porcine alveolar macrophages (PAMs), and PRRSV infection downregulated MARCO expression both *in vitro* and *in vivo*. MARCO was not involved in viral adsorption and internalization processes, indicating that MARCO may not be a PRRSV-entry facilitator. Contrarily, MARCO served as a host restriction factor for PRRSV. The knockdown of MARCO in PAMs enhanced PRRSV proliferation, whereas overexpression suppressed viral proliferation. The N-terminal cytoplasmic region of MARCO was responsible for its inhibitory effect on PRRSV. Further, we found that MARCO was a proapoptotic factor in PRRSV-infected PAMs. MARCO knockdown weakened virus-induced apoptosis, whereas overexpression aggravated apoptosis. MARCO aggravated GP5-induced apoptosis, which may result in its proapoptotic function in PAMs. The interaction between MARCO and GP5 may contribute to the intensified apoptosis induced by GP5. Additionally, the inhibition of apoptosis during PRRSV infection weakened the antiviral function of MARCO, suggesting that MARCO inhibits PRRSV through the regulation of apoptosis. Taken together, the results of this study reveal a novel antiviral mechanism of MARCO and suggest a molecular basis for the potential development of therapeutics against PRRSV.

**IMPORTANCE** Porcine reproductive and respiratory syndrome virus (PRRSV) has been one of the most serious threats to the global swine industry. Glycoprotein 5 (GP5) exposed on the surface of PRRSV virions is a major glycoprotein, and it is involved in viral entry into host cells. A macrophage receptor with collagenous structure (MARCO), which is a member of the scavenger receptor family, was identified to interact with PRRSV GP5 in a DUALmembrane yeast two-hybrid screening. Further investigation demonstrated that MARCO may not serve as a potential receptor to mediate PRRSV entry. Instead, MARCO was a host restriction factor for the virus, and the N-terminal cytoplasmic region of MARCO was responsible for its anti-PRRSV effect. Mechanistically, MARCO inhibited PRRSV infection through intensifying virus-induced apoptosis in PAMs. The interaction between MARCO and GP5 may contribute to GP5-induced apoptosis. Our work reveals a novel antiviral mechanism of MARCO and advances the development of control strategies for the virus.

## INTRODUCTION

Porcine reproductive and respiratory syndrome virus (PRRSV) is an enveloped, single-stranded, positive-sense RNA virus that causes diseases ranging from late-term abortions and stillbirths in sows to respiratory diseases in piglets and growing pigs (1, 2). Since the initial outbreak in the late 1980s in North America and Europe, PRRSV has been one of the most economically devastating pathogens for the global swine industry. The genome of PRRSV is proximately 15 kb in length, and it is organized with 10 open reading frames (ORFs). The 5′-proximal large ORF1a and ORF1b constitute almost two-thirds of the whole-genome, and they encode two polyproteins, namely, pp1a and pp1ab (3). These two polyproteins are further cleaved into individual 14 nonstructural proteins (nsps), and these are required for PRRSV genome RNA synthesis or for antagonizing host antiviral responses (4, 5). The remaining ORFs 2 to 7, which are situated in the 3′-end, encode 8 structural proteins, including the nucleocapsid protein N, the three nonglycosylated membrane proteins M, E, and ORF5a, as well as the four membrane-associated glycoproteins GP2a, GP3, GP4, and GP5 (2, 6). GP5 is a major glycoprotein that is exposed on the surface of PRRSV virions, is involved in viral entry into host cells, and is a main target for neutralizing antibodies (7). Moreover, GP5 has repeatedly been reported to be a cause of PRRSV-induced apoptosis (8, 9). GP2a, GP3, and GP4 are all minor envelope proteins that form heterotrimers via disulfide linkage in virions to mediate the receptor binding, similar to GP5 (10).

PRRSV has a highly restricted host tropism and prefers to infect the well-differentiated cells of the monocyte/macrophage lineage, particularly porcine alveolar macrophages (PAMs) (11, 12). PAMs and blood monocytes are the only porcine cells that effectively support viral propagation (13). Nonporcine Marc-145, which is a subclone of the monkey kidney cell line MA104, is fully permissive for PRRSV replication and has become a useful tool for PRRSV research *in vitro* (14). Viral entry into host cells is mandatory for infection. The binding of certain glycoproteins or structures on the virus particle surface to particular cell receptors gives rise to viral penetration into target cells or to the fusion of the viral envelope with the host cell membrane and the following viral genomic release (15). The binding of heparin sulfate to the M/GP5 complex of PRRSV contributes to the concentration of virions on the cell surface (16, 17). Scavenger receptor CD163 interacts with the GP2a/GP3/GP4 complex to mediate membrane fusion (13, 18, 19). It is the interactions between CD163 and these minor glycoproteins that may determine the cell and host tropisms of PRRSV. In the face of the scarcity of available vaccines, the interactions between PRRSV glycoproteins and host membrane receptors have become extremely attractive intervention points at which to impede PRRSV infection.

To identify more potential PRRSV entry facilitators, a split-ubiquitin yeast two-hybrid (DUALmembrane Y2H) screening was performed. Using PRRSV GP5 as a bait, the macrophage receptor with collagenous structure (MARCO), which is a scavenger receptor family member, was identified. Like the CD163 molecule, MARCO is highly expressed in lung tissues, but it is only on specific subsets of macrophages (20). MARCO is appreciated for its role in sensing and clearing pathogens through the recognition of pathogen-associated molecular patterns (PAMPs) (21). The ligands that are recognized by MARCO are diverse and are often polyanionic in nature, including inorganic particulates, nucleic acids, bacterial lipopolysaccharides, viruses, oxidized lipoproteins, as well as modified endogenous proteins, dead cells, or debris (22). Upon MARCO binding to the ligand on the cell surface, the receptor-ligand complex is delivered to the endosomes through clathrin-coated vesicles, after which the ligand is released, and MARCO is recycled to the plasma membrane (23). As a scavenger receptor, MARCO is routinely thought to be conducive to the host antiviral defense. Nevertheless, it is occasionally usurped by viruses. Until now, herpes simplex virus 1, adenovirus, and vaccinia virus have been reported to exploit MARCO to promote cell surface adsorption (24–26). Although it is termed as a scavenger, MARCO does more than phagocytosis. MARCO functions in cellular adhesion, migration, and antigen presentation (27–29). The facts that MARCO interacts with PRRSV GP5 in a Y2H screening and that MARCO has extensive similarities to the receptor of CD163 galvanizes our interest in whether MARCO is involved in the process of PRRSV infection.

In this study, we found that MARCO was constitutively expressed in PAMs and was downregulated upon PRRSV infection. Although MARCO was confirmed to interact with PRRSV GP5, it affected neither viral adsorption nor the internalization process, suggesting that MARCO may not serve as a receptor for PRRSV. In contrast, we demonstrated that MARCO was an anti-PRRSV factor. The knockdown of MARCO in PAMs enhanced PRRSV reproduction, whereas overexpression suppressed PRRSV reproduction. The N-terminal cytoplasmic region of MARCO was responsible for its inhibitory effect on PRRSV, and, interestingly, this domain affected the location of MARCO on the cell membrane. Further, we found that MARCO exacerbates PRRSV-induced apoptosis, and the interaction between MARCO and GP5 may contribute to its proapoptotic function. The inhibition of apoptosis during infection weakened the inhibition of MARCO on PRRSV, suggesting that MARCO restricts PRRSV infection through the regulation of apoptosis.

## RESULTS

### MARCO is identified as one of the host factors that interacts with PRRSV GP5.

**FIG 1:**
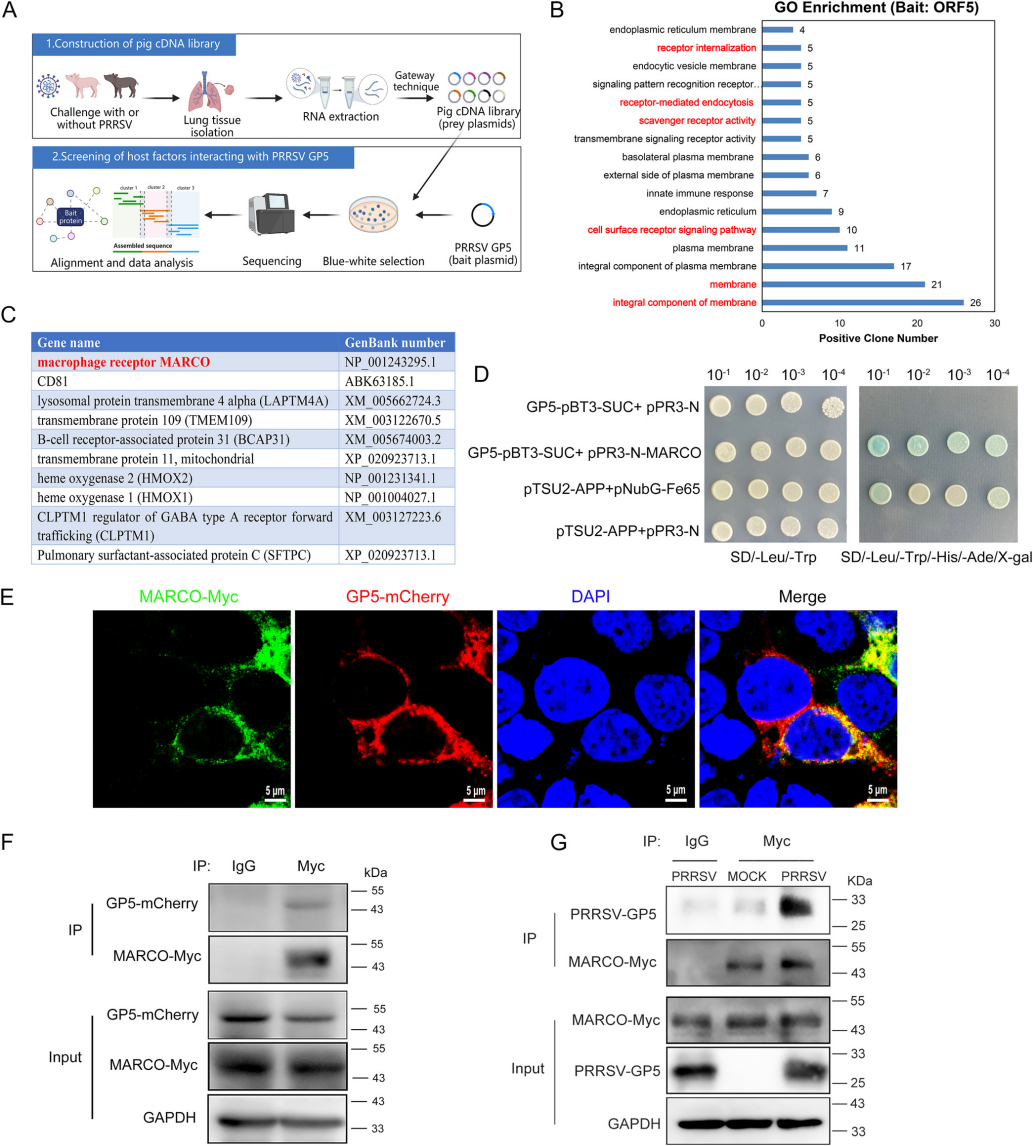
MARCO is identified as one of the host factors that interacts with PRRSV GP5. (A) Schematic of porcine cDNA library construction and yeast two-hybrid screening using PRRSV GP5 as a bait. The lung tissue samples from piglets challenged with or without PRRSV were pooled, and total RNA was subsequently extracted. Double-stranded cDNA was synthesized and cloned into each of three reading frames of the pDONR222 plasmid, and this was followed by recombination with the pPR3-N-DEST vector based on gateway DNA recombinant technology to obtain an expressed yeast porcine cDNA library (upper panel). A DUALmembrane Y2H screening was performed to identify potential host factors interacting with PRRSV GP5. PRRSV GP5 was cloned into the pBT3-SUC vector to generate the bait plasmid GP5-pBT3-SUC. The cDNA library was cloned into the pPR3-N-DEST vector to generate prey plasmids. All prey plasmids, together with the GP5-pBT3-SUC bait plasmid, were cotransformed into the yeast strain Y2HGold. The positive colonies were analyzed via DNA sequencing, and candidates interacting with PRRSV GP5 were identified via BLAST searches of the NCBI database (lower panel). (B) The gene ontology (GO) enrichment analysis of the candidate proteins that interact with PRRSV GP5. (C) The 10 selected interest proteins, including MARCO (marked red) from 49 candidates. (D) Confirmation of interaction between MARCO and PRRSV GP5, using Y2H for testing. The yeast strain NMY51 was cotransformed with the bait plasmid GP5-pBT3-SUC and the prey plasmid pPR3-N-MARCO. The cotransformation of pTSU2-APP/pNubG-Fe65 and GP5-pBT3-SUC/pPR3-N or pTSU2-APP/pPR3-N were used as the positive and negative controls, respectively. The transformants were grown on synthetic dropout (SD) medium. The interaction between GP5 and MARCO was identified by the growth of colonies on SD/-Leu/-Trp/-His/-Ade plates. SD/-Leu/-Trp/-His/-Ade, SD agar medium without leucine, tryptophan, histidine, and adenine; SD/-Leu/-Trp, SD agar medium without leucine and tryptophan. (E) The colocalization of the MARCO protein with PRRSV GP5 in 293T cells. The cells were cotransfected with Myc-tagged MARCO and mCherry-tagged GP5 plasmids for 24 h. The cells were fixed and immunostained with the anti-Myc antibody. The nuclei were stained with DAPI. Fluorescent images were acquired using laser-scanning fluorescent confocal microscopy. Bar, 5 μm. (F) Confirmation of interaction between MARCO and PRRSV GP5 using a co-IP assay. 293T cells were cotransfected with mCherry-tagged GP5 and Myc-tagged MARCO plasmids for 24 h. The cells were then lysed, and an IP analysis was performed using a mouse anti-Myc antibody. (G) The interaction between MARCO and PRRSV GP5 in the case of a PRRSV infection. Marc-145 cells were transiently transfected with the Myc-tagged MARCO plasmid for 24 h, prior to a PRRSV infection. At 24 hpi, the cells were harvested for an IP analysis using the anti-Myc antibody.

The CD163 receptor of PRRSV mediates the merging of the PRRSV lipid envelope with the endosome membrane through interacting with the virus surface GP2a/GP3/GP4 complex in PAMs or Marc-145 cells, which is mandatory for the subsequent viral genomic release. GP5 is a major glycoprotein of PRRSV. However, whether and how the interactions between PRRSV GP5 and host proteins affect the viral infection process have not been well-studied. To identify the potential host factors that interact with PRRSV GP5, a porcine cDNA library was first constructed for Y2H screening using PRRSV-infected and uninfected lung tissues. The cDNA library prey plasmids, together with a viral GP5 bait plasmid, were cotransformed into the yeast strain Y2HGold, and host proteins interacting with the viral GP5 were screened out (Fig. 1A). The library screening yielded 66 positive colonies and identified 49 potential interactors. A gene ontology (GO) enrichment analysis showed that these candidates were mainly located on the plasma membrane or endoplasmic reticulum membrane and functioned in receptor internalization, scavenger receptor activity, or receptor-mediated endocytosis (Fig. 1B), indicating that the potential interactors are membrane-associated proteins and may play a role in mediating virus entry. We subsequently selected 10 interest proteins from these candidates, based on the corresponding number of positive colonies (Fig. 1C). Among them, MARCO, a member of the scavenger receptor family that has extensive similarities to the receptor of CD163, was chosen. The following study focused on the behavior of MARCO during PRRSV infection.

We further verified the bait-prey pair of GP5-MARCO in a pairwise Y2H testing. The bait plasmid GP5-pBT3-SUC and prey plasmid pPR3-N-MARCO were cotransformed into yeast strain NMY51. The growth of blue colonies on the synthetic quadruple dropout medium, supplemented with X-gal, indicated a positive interaction between MARCO and GP5 (Fig. 1D). To further observe the interaction between viral GP5 and MARCO in mammalian cells, 293T cells were cotransfected with mCherry-tagged GP5 and Myc-tagged MARCO plasmids for 24 h. The confocal microscopy assay showed a prominent colocalization of MARCO and GP5 in 293T cells (Fig. 1E). Additionally, mCherry-tagged GP5 was coprecipitated with Myc-tagged MARCO in the coimmunoprecipitation (co-IP) assay in 293T cells (Fig. 1F). In PRRSV-infected Marc-145 cells, PRRSV GP5 was especially pulled down by the anti-Myc antibody, suggesting an interaction between PRRSV GP5 and MARCO in cases of infection (Fig. 1G). Together, these results suggest that MARCO interacts with PRRSV GP5.

### PRRSV infection downregulates MARCO expression *in vivo* and *in vitro*.

**FIG 2:**
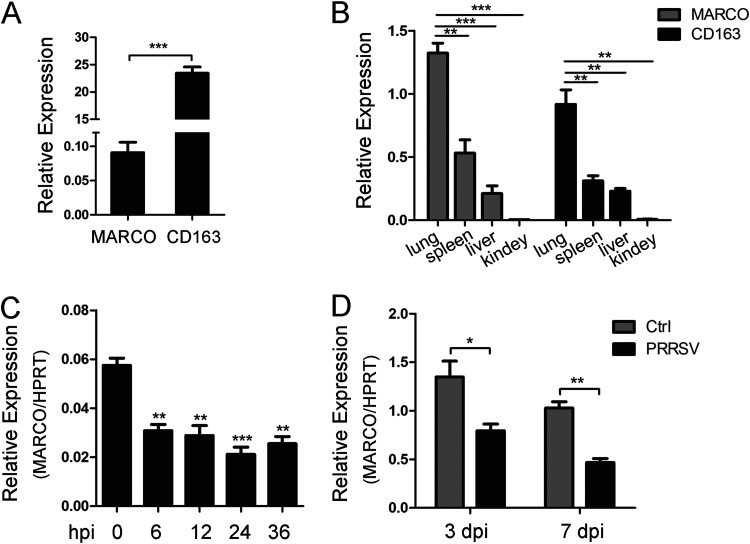
PRRSV infection downregulates MARCO expression *in vivo* and *in vitro*. (A) The mRNA level of MARCO and CD163 in PAMs was detected via RT-qPCR. (B) The mRNA level of MARCO and CD163 in various tissues. Different tissues were separated from 4- to 6-week-old PRRSV-negative, healthy piglets, and total RNA was extracted to perform a RT-qPCR analysis. (C) The transcriptional level change of MARCO in PAMs during PRRSV infection. The PAMs were mock-infected or were infected with PRRSV at a multiplicity of infection (MOI) of 0.5 for various times. At the indicated time points, the PAMs were harvested for RT-qPCR. (D) The transcriptional level change of MARCO in the lungs of PRRSV-infected pigs. PRRSV-negative piglets were challenged with PRRSV at 4 to 6 weeks of age. On days 3 and 7 postchallenge, three piglets from the infected and control group were euthanized, and their lung tissues were collected to perform the RT-qPCR analysis. HPRT, the hypoxanthine phosphoribosyltransferase of Sus scrofa, was used as an endogenous reference in RT-qPCR. The data are represented as the mean ± SE of the three biological replicates. Significant differences are indicated as: *, *P* < 0.05; **, *P* < 0.01; and ***, *P* < 0.001.

MARCO is restricted to macrophages. Exactly, MARCO is exclusively expressed in the macrophages of certain tissues, such as spleen marginal zone peritoneal macrophages, medullary cords macrophages, and peritoneal macrophages (20, 25). Here, we that found MARCO was present in PAMs, but they were present at a much lower level than that of the virus receptor CD163 (Fig. 2A). As expected, similar to CD163, MARCO was most highly expressed in the lung tissues of pigs (Fig. 2B). To investigate whether MARCO was involved in PRRSV infection, we first examined the effect of PRRSV on MARCO expression. Upon a PRRSV strain Li11 infection, the mRNA level of MARCO in PAMs was reduced by half at various hours postinfection (hpi) (Fig. 2C). The decreased MARCO expression that was induced by PRRSV was further confirmed in pigs that were challenged with PRRSV *in vivo*. On days 3 and 7 postchallenge, the transcriptional level of MARCO in the lungs of the infected animals was twice as low as that observed in null-infected pigs (Fig. 2D). Together, these results illustrate that PRRSV downregulates MARCO expression *in vivo* and *in vitro*.

### MARCO may not be involved in the entry of PRRSV into PAMs.

**FIG 3:**
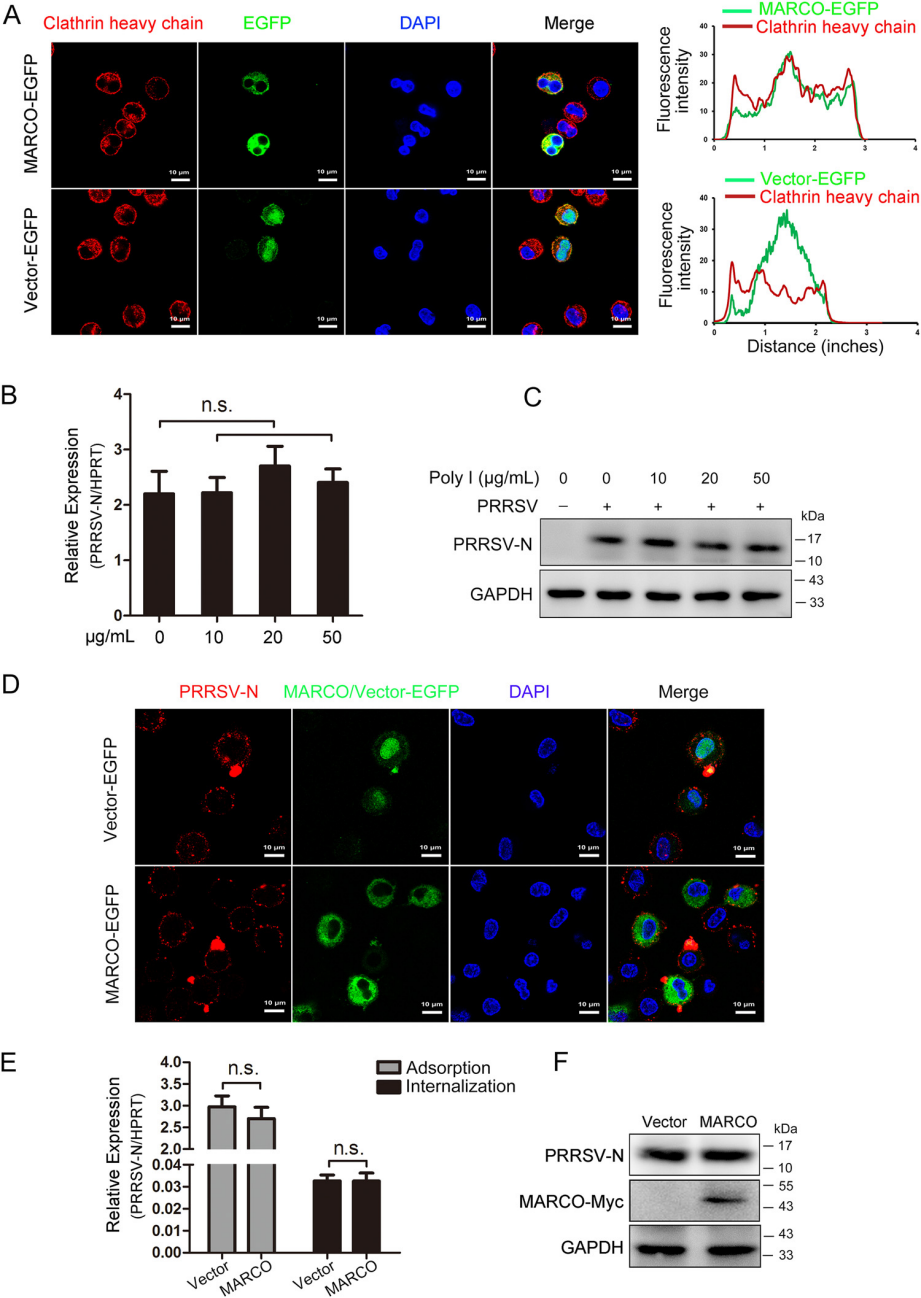
MARCO may not be involved in the entry of PRRSV into PAMs. (A) Route of MARCO internalization. PAMs were infected with recombinant adenovirus for 24 h to express MARCO-EGFP and were incubated with MARCO ligand Dxs (50 μg/mL, Sigma, D8787) at 37°C for 30 min. The cells were fixed for the immunostaining of clathrin heavy chain. The nuclei were stained with DAPI. Bar, 10 μm. Colocalization quantifications were done using the Plot Profile plugin in ImageJ. (B and C) Effects of MARCO ligand Poly(I) on PRRSV adsorption. PAMs were incubated with Poly(I) (Sigma, P4154) at 37°C for 30 min, and this was followed by PRRSV incubation at a MOI of 5 at 4°C for 2 h. After washing with cold PBS three times to remove the unbound virions, PAMs were collected for RT-qPCR and Western blotting analyses to detect the mRNA (panel B) and protein (panel C) levels of PRRSV N on the surfaces of the PAMs. (D–F) Effects of MARCO overexpression on PRRSV adsorption and internalization. PAMs infected with recombinant adenovirus to overexpress MARCO were incubated with PRRSV at a MOI of 5 at 4°C for 2 h and were washed with cold PBS to remove the unbound virions. Adsorbed PRRSV was detected using immunofluorescence staining (panel D), RT-qPCR (panel E), or Western blotting (panel F). Bar, 10 μm in the immunofluorescence analysis. For the internalized PRRSV, the PAMs were switched to 37°C for another 1 h after the removal of the unbound virus. RT-qPCR was performed to detect the mRNA level of PRRSV N in the PAMs (E). HPRT, the hypoxanthine phosphoribosyltransferase of Sus scrofa, was used as an endogenous reference in the RT-qPCR. The data are represented as the mean ± SE of three biological replicates. n.s. indicates no difference.

Entry into cells is the first step of the virus life cycle. The virus first attaches to and then penetrates into the host target cells. Clathrin-mediated endocytosis is the major route that is used by PRRSV for entry into PAMs or Marc-145 cells (30). As a scavenger, MARCO is internalized into endosomes from the cell surface upon the recognition of its ligand. We first examined the trafficking of MARCO in PAMs through an immunofluorescence analysis. PAMs were infected with recombinant adenovirus to transiently express MARCO-EGFP and were then incubated with ligand dextran sulfate (Dxs) at 37°C for 30 min. Based on the fluorescence intensity plot profile analysis, confocal microscopy revealed a striking colocalization of MARCO-EGFP with a clathrin heavy chain in the presence of Dxs (Fig. 3A). This suggests that, like PRRSV, MARCO is internalized through clathrin-mediated endocytosis in PAMs. We next investigated the role of MARCO in PRRSV entry. PAMs were treated with another ligand, namely, Poly(I), which a single-stranded RNA component of Poly (I:C), at 37°C for 30 min before infection with PRRSV at a MOI of 5. The cells were incubated at 4°C for 2 h to allow for PRRSV adsorption. After removing the unbound virions, the level of PRRSV N on the cell surface was detected. Neither the mRNA nor the protein levels of PRRSV N were affected by the Poly(I) treatment (Fig. 3B and C). To unequivocally assess the impact of MARCO on PRRSV adsorption, we detected the level of PRRSV N, and this was followed by the overexpression of MARCO in PAMs, using recombinant adenovirus. The immunostaining of PRRSV N exhibited comparable levels of virions on MARCO-overexpressing PAMs and on the control PAMs (Fig. 3D). The RT-qPCR and Western blotting analyses further confirmed the results (Fig. 3E and F). Moreover, we found that MARCO overexpression in PAMs had no effects on the internalization of PRRSV virions (Fig. 3E). Taken together, these findings indicate that MARCO may not be involved in the entry process of PRRSV, even though MARCO interacts favorably with PRRSV GP5.

### MARCO is a host restriction factor for PRRSV.

**FIG 4:**
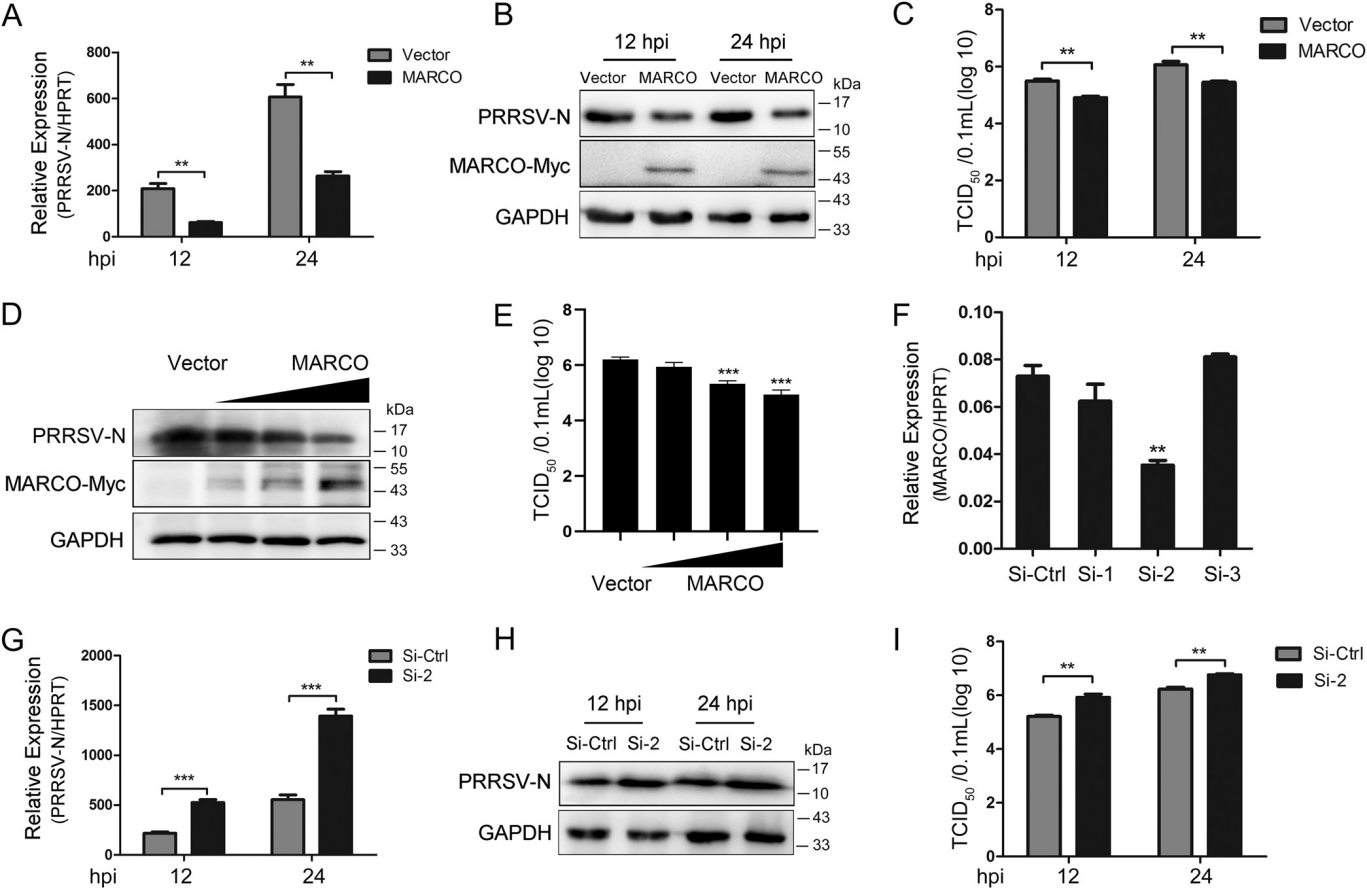
MARCO is a host restriction factor for PRRSV. (A–E) Effects of MARCO overexpression on PRRSV proliferation in PAMs. PAMs were infected with MARCO recombinant adenovirus for 24 h, and this was followed by infection with PRRSV at a MOI of 0.5. Cells were harvested at 12 or 24 hpi for RT-qPCR and Western blotting to examine the mRNA (A) and protein levels (B) of PRRSV N. (C) PAMs were infected with MARCO recombinant adenovirus for 24 h, and this was followed by infection with PRRSV at a MOI of 0.5. At 12 or 24 hpi, the cell supernatant was collected to determine the virus titers via a TCID_50_ assay. (D and E) PAMs were infected with different doses of MARCO recombinant adenovirus for 24 h and were then infected with PRRSV at a MOI of 0.5. At 24 hpi, the cells were lysed to detect the PRRSV N (panel D), and the corresponding supernatant was collected to examine the virus titers using a TCID_50_ assay (panel E). (F–I) Effects of MARCO knockdown on PRRSV proliferation in PAMs. (F) PAMs were transiently transfected with three independent small interfering RNAs (siRNA) targeting MARCO for 24 h and were harvested for RT-qPCR to examine the silencing efficiency of MARCO. (G–I) PAMs were transiently transfected with siRNA2 for 24 h, and this was followed by infection with PRRSV at a MOI of 0.5. At 12 or 24 hpi, cells were harvested to determine the PRRSV N mRNA (panel G) and protein (panel H) levels via RT-qPCR and Western blotting. The cell supernatant was collected to examine the virus titers via a TCID_50_ assay (panel I). HPRT, the hypoxanthine phosphoribosyltransferase of Sus scrofa, was used as an endogenous reference in the RT-qPCR. The data are represented as the mean ± SE of three biological replicates. Significant differences are indicated as: **, *P* < 0.01 and ***, *P* < 0.001.

Although MARCO did not participate in PRRSV entry, we further explored the role of this protein in the subsequent infectious events, given its multifunctional potential. PAMs were infected with recombinant adenovirus to exogenously express MARCO prior to infection with PRRSV at a MOI of 0.5 for various times. Notably, the mRNA and protein levels of PRRSV N at 12 and 24 hpi were consistently reduced in MARCO-overexpressing PAMs, compared to those in control cells (Fig. 4A and B). The impaired PRRSV proliferation was further verified by the lower viral titers in the supernatants of MARCO-overexpressing PAMs (Fig. 4C). Moreover, exogenously expressed MARCO by recombinant adenovirus inhibited PRRSV N and viral titers in PAMs in a dose-dependent manner (Fig. 4D and E). To further investigate the function of endogenous MARCO on PRRSV, PAMs were transiently transfected with three independent small interfering RNAs (siRNAs) to silence MARCO expression. As shown in Fig. 4F, si-2 efficiently weakened the transcript level of endogenous MARCO in PAMs at 24 h posttransfection. We found that MARCO knockdown significantly elevated the expression of PRRSV N at both the mRNA and protein levels in PAMs at 12 and 24 hpi (Fig. 4G and H). Meanwhile, a higher virus titer was observed in the supernatants of PAMs with MARCO knockdown (Fig. 4I). Taking these results together, we conclude that MARCO serves as a host restriction factor during PRRSV infection.

### The N-terminal intracellular domain of MARCO, which affects its location on the cell membrane, is responsible for its inhibitory effect on PRRSV.

**FIG 5:**
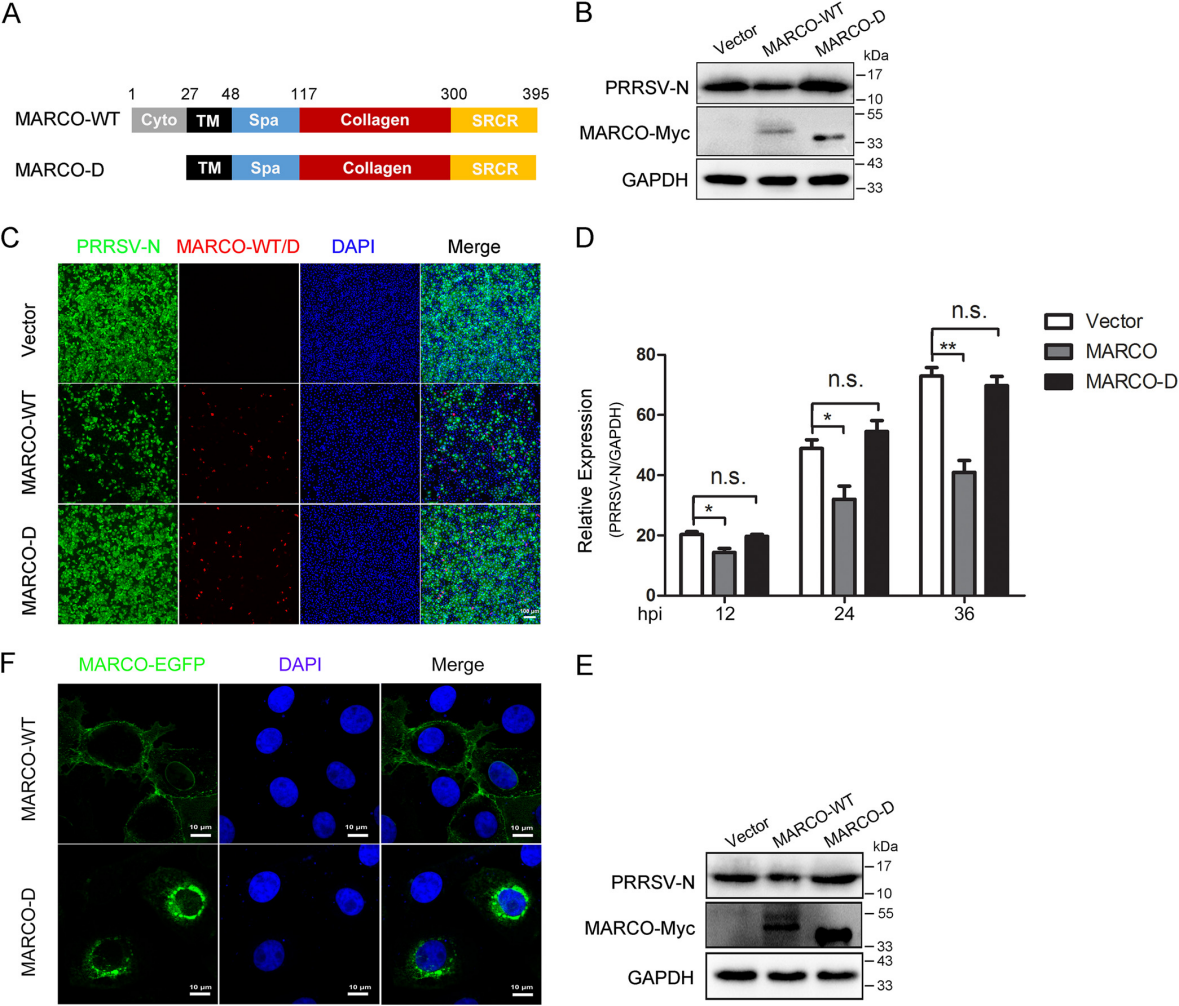
The N-terminal intracellular domain of MARCO is required for its inhibitory effect on PRRSV. (A) Schematic representations of full-length or truncated MARCO constructs. Cyto, cytoplasmic domain; TM, transmembrane domain; Spa, spacer domain; collagen, collagenous domain; SRCR, scavenger receptor cysteine-rich domain. (B) PAMs were exogenously infected with recombinant adenovirus to express MARCO-WT or MARCO-D with N-terminal cytoplasmic domain deletion for 24 h, and they were infected with PRRSV at a MOI of 0.5 for 24 h. Cells were harvested for Western blotting to detect the PRRSV N level. (C–E) Marc-145 cells were transiently transfected with Myc-tagged MARCO-WT or MARCO-D plasmid for 24 h, and they were infected with PRRSV at a MOI of 0.5. At 24 hpi, immunofluorescence (C), RT-qPCR (D), and Western blotting (E) analyses were performed to examine the PRRSV N levels. Bar, 100 μm. (F) Marc-145 cells were infected with recombinant adenovirus to express MARCO-WT-EGFP or MARCO-D-EGFP heterogeneously for 24 h. Cells were fixed, and this was followed by nuclei staining with DAPI. Images of cells were acquired via laser-scanning fluorescent confocal microscopy. Bar, 10 μm. GAPDH, the glyceraldehyde-3-phosphate dehydrogenase of Chlorocebus sabaeus, was used as an endogenous reference in RT-qPCR. The data are represented as the mean ± SE of the three biological replicates. Significant differences are indicated as: *, *P* < 0.01 and **, *P* < 0.05.

MARCO is a type II transmembrane glycoprotein, and it structurally contains a short intracellular and transmembrane domain as well as a large extracellular domain that is composed of a spacer domain, a long collagenous domain, and a C-terminal scavenger receptor cysteine-rich domain (SRCR) (Fig. 5A) (31). The SRCR domain is required for ligand binding, and the collagenous domain contributes to the homotrimer formation (31, 32). Although the intracellular part of MARCO is short (species-dependent, residues 1 to 26 in pig MARCO), it is predicted to be associated with membrane trafficking and recycling, cell adhesion, and signal transduction (33, 34). To determine which domain is required for the inhibitory effect of MARCO on PRRSV, we constructed a series of MARCO truncation mutants (Fig. 5A). Unexpectedly, the mutants that are devoid of the SRCR domain or the collagenous domain could not successfully express in eukaryotic cells (data not shown), suggesting the functional importance of these two domains for pig MARCO expression. The cytoplasmic domain deleted mutant (MARCO-D) was expressed comparably with wild-type MARCO (MARCO-WT) in PAMs (Fig. 5B). PAMs were infected with PRRSV at a MOI of 0.5 for 24 h after infection with MARCO-WT or MARCO-D recombinant adenovirus. Western blotting data showed that the exogenous expression of MARCO-D completely abrogated the inhibitory effect of MARCO on PRRSV, indicating that the cytoplasmic domain of MARCO is responsible for the anti-PRRSV property. To further characterize the antiviral function of the cytoplasmic domain of MARCO, a Myc-tagged MARCO-WT or MARCO-D plasmid was transiently transfected into Marc-145 cells in which MARCO was absent. The exogenous expression of the pig MARCO truncated mutant in Marc-145 cells could avoid the interferences of endogenous proteins. Consistent with the findings in PAMs, the immunofluorescence analysis found that, compared with the control vector, the exogenous expression of MARCO-WT obviously inhibited PRRSV proliferation in Marc-145 cells, whereas the exogenous expression of MARCO-D showed no effects on PRRSV (Fig. 5C). The phenomenon was also demonstrated in both RT-qPCR and Western blotting analyses (Fig. 5D and E). The cytoplasmic region of MARCO is predicted to be associated with its trafficking. Interestingly, the confocal microscopy results showed that the exogenous pig MARCO-WT was mainly located on the cell surface; however, a large proportion of the exogenous MARCO-D existed in intracellular pools, suggesting an essential role of the cytoplasmic domain in the subcellular localization of MARCO (Fig. 5F). In conclusion, these results indicate that the cytoplasmic region is responsible for the suppressive role of MARCO on PRRSV, and this region affects the distribution of MARCO on the cellular membrane.

### The interaction between MARCO and PRRSV GP5 may aggravate PRRSV-induced apoptosis, which contributes to the inhibition function of MARCO on PRRSV.

**FIG 6:**
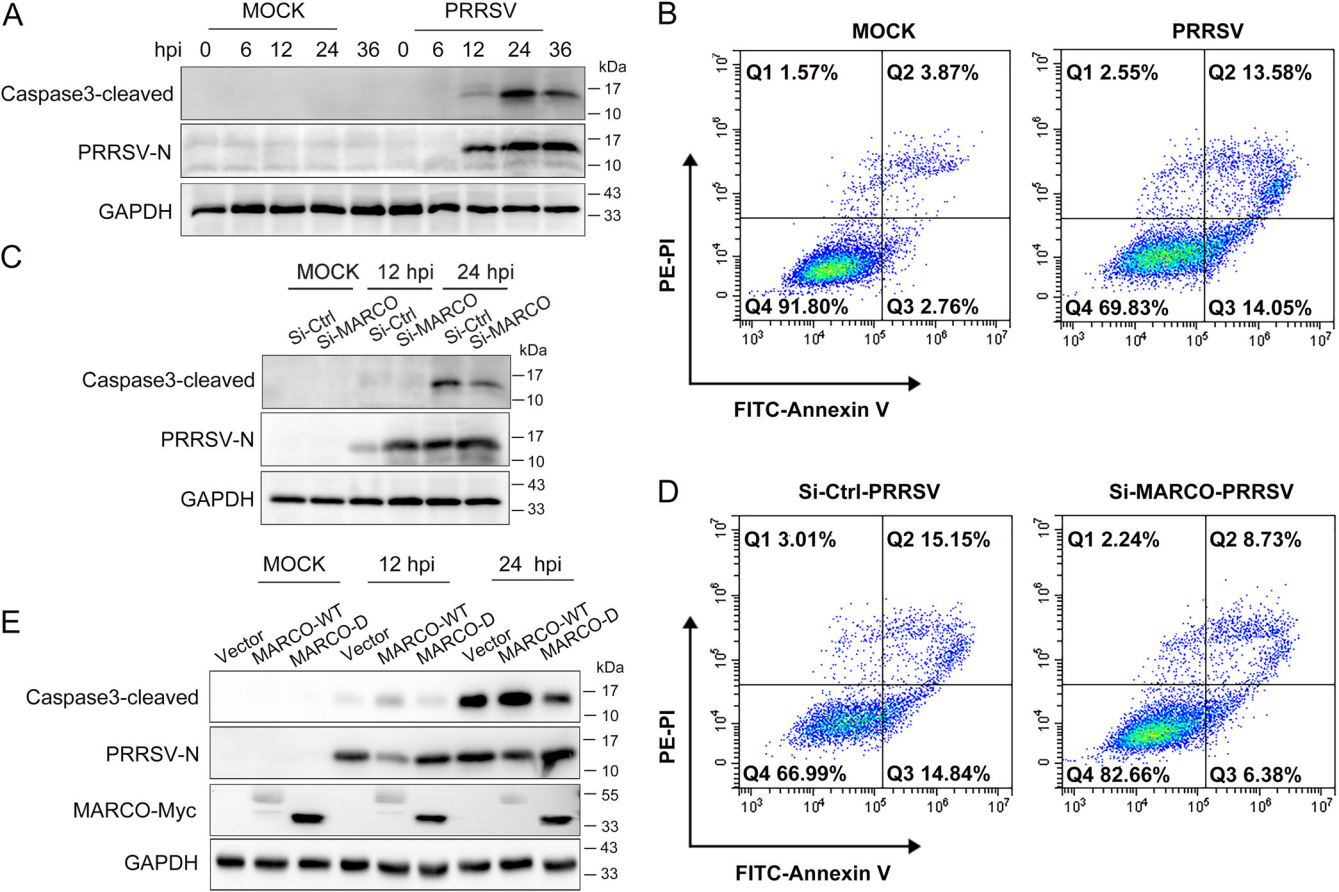
MARCO is a proapoptotic factor in PRRSV-infected PAMs. (A) PAMs were mock-infected or infected with PRRSV at a MOI of 0.5 for various times, and cells were lysed for Western blotting to determine the levels of cleaved caspase3 and PRRSV N. (B) PAMs were mock-infected or infected with PRRSV at a MOI of 0.5 for 24 h, and cells were digested for an Annexin-V/PI double staining analysis. (C) PAMs were transiently transfected with siRNA2 for 24 h and were then mock-infected or infected with PRRSV at a MOI of 0.5. At 12 or 24 hpi, cells were harvested to determine the levels of cleaved caspase3 and PRRSV N. (D) PAMs were transiently transfected with siRNA2 for 24 h and were then infected with PRRSV at a MOI of 0.5. At 24 hpi, cells were digested for Annexin-V/PI double staining analysis. (E) PAMs were infected with recombinant adenovirus to express MARCO-WT or MARCO-D exogenously for 24 h and were then mock-infected or infected with PRRSV at a MOI of 0.5 for 24 h. The cells were harvested for Western blotting to detect the levels of cleaved caspase3 and PRRSV N.

Apoptosis, which is programmed cell death, is a common outcome of virus infection. Despite claims to the contrary, PRRSV has been documented to induce PAMs apoptosis (35). Furthermore, accumulating studies suggest that MARCO is involved in cell apoptosis (36–39). Therefore, it is conceivable that MARCO may be involved in the regulation of PRRSV-induced apoptosis. First, we examined the activation of apoptosis executioner caspase3 upon PRRSV infection. PAMs were mock-infected or infected with PRRSV at a MOI of 0.5 for various times. Western blotting data revealed that the cleavage of caspase3 occurred at 12 hpi and was most intense at 24 hpi in PAMs (Fig. 6A). Moreover, an Annexin-V/PI apoptosis analysis, using flow cytometry, showed that the proportion of early and late apoptotic cells was almost negligible in the mock-infected control culture; however, at 24 hpi, the percentages of early and late apoptotic cells increased to 14.05% and 13.58%, respectively (Fig. 6B). These results indicate that PRRSV infection triggers apoptosis in PAMs. Next, we investigated whether MARCO is involved in PRRSV-induced apoptosis. MARCO was knocked down in PAMs before PRRSV infection. Western blotting revealed that the level of cleaved caspase3 was obviously reduced in PAMs with MARCO knockdown at both 12 and 24 hpi (Fig. 6C). Furthermore, the Annexin-V/PI double staining showed that the percentages of early and late apoptotic cells were decreased by 8.46% and 6.42%, respectively, in MARCO-silencing PAMs, compared to those in the controls (Fig. 6D). On the contrary, we found that MARCO-WT, not MARCO-D overexpression, elevated the level of cleaved caspase3 in PAMs (Fig. 6E). These results indicate that MARCO is a proapoptotic factor in PRRSV-infected PAMs and that the cytoplasmic domain of MARCO is important for the induction of apoptotic signals.

**FIG 7:**
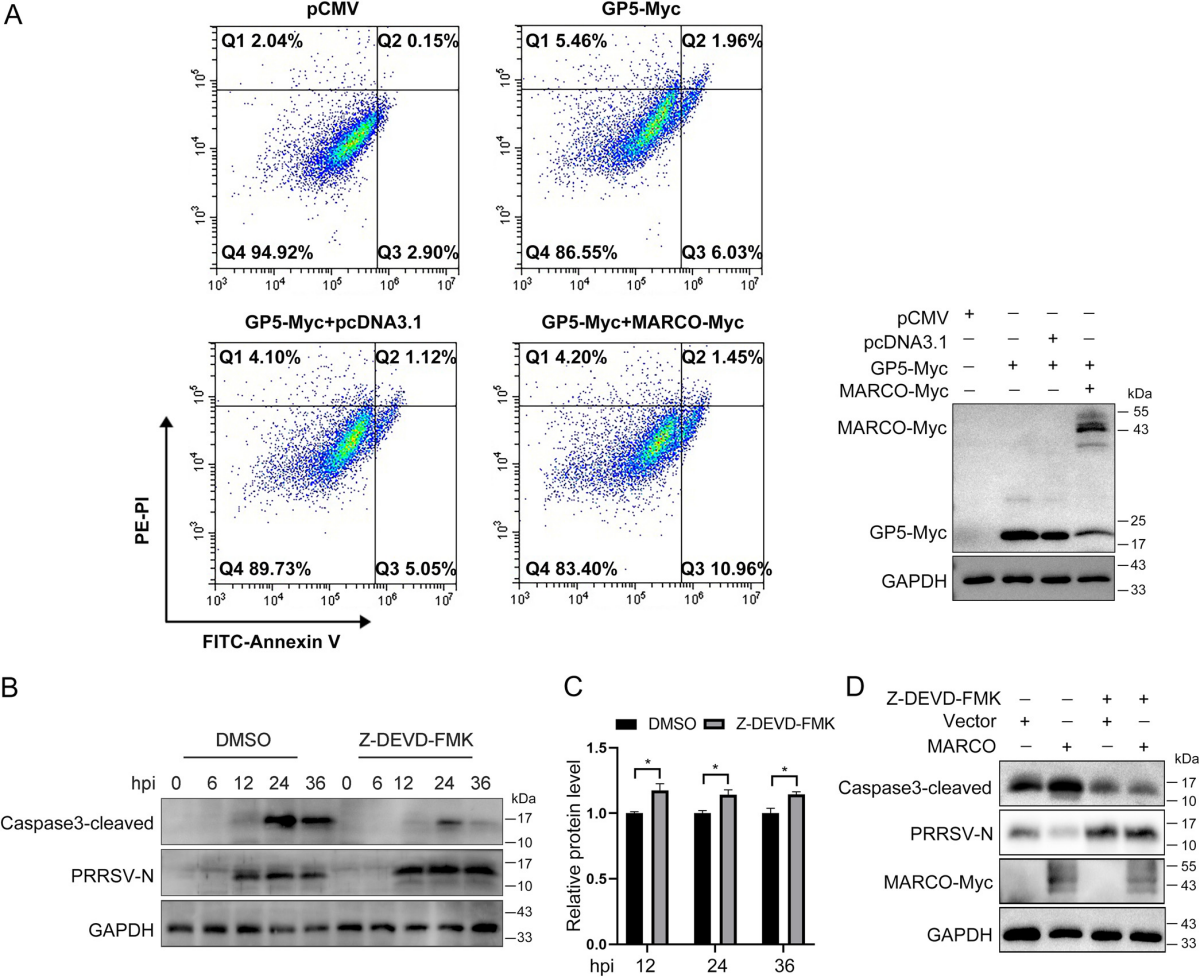
The interaction between MARCO and PRRSV GP5 may aggravate PRRSV-induced apoptosis, which contributes to the inhibition function of MARCO on PRRSV. (A) PRRSV GP5 is an inducer of apoptosis, and MARCO overexpression further aggravates apoptosis. Marc-145 cells were transiently transfected with an empty vector, transfected with an GP5-expressing plasmid, or cotransfected with plasmids encoding GP5 and MARCO or an empty vector for 24 h. The cells were digested for a flow cytometry analysis of the apoptosis. The expression of Myc-tagged GP5 and Myc-tagged MARCO in the Marc-145 cells was examined via Western blotting. (B) PAMs were infected with PRRSV at a MOI of 0.5 for various times in the presence or absence of the caspase3 inhibitor Z-DEVD-FMK (30 μM, Abmole, 210344-95-9). The cells were lysed for Western blotting to determine the levels of cleaved caspase3 and PRRSV N. (C) The quantization of the PRRSV N protein abundance in panel B. (D) The PAMs were infected with recombinant adenovirus for 24 h to overexpress MARCO, and this was followed by infection with PRRSV at a MOI of 0.5 in the presence or absence of Z-DEVD-FMK (30 μM). At 24 hpi, the PAMs were lysed to examine the levels of PRRSV N and cleaved caspase3 via Western blotting.

It has been reported that the GP5 of PRRSV is a cause of virus-induced apoptosis (8). Having demonstrated that MARCO interacts with GP5 and that MARCO intensifies apoptosis induced by PRRSV in PAMs, we speculated that the interaction between MARCO and GP5 may be involved in the proapoptotic function of MARCO. To prove this hypothesis, Marc-145 cells were transiently transfected with an empty vector, transfected with a GP5-expressing plasmid, or cotransfected with plasmids encoding GP5 and MARCO or an empty vector for 24 h. The Annexin-V/PI apoptosis analysis showed that the cells were obviously classified into two groups and that the percentages of early and late apoptotic cells were increased to 6.03% and 1.96%, respectively, in the GP5 expression group, compared to those in the empty vector control group, confirming the previous report that the GP5 of PRRSV is an inducer of apoptosis (Fig. 7A, upper panel). In addition, we found that MARCO expression further increased the proportion of FITC-positive cells, suggesting that MARCO accelerates the early apoptosis induced by GP5 in Marc-145 cells (Fig. 7A, lower panel). Therefore, we conclude that the interaction between MARCO and GP5 may contribute to the proapoptotic function of MARCO.

Classically, proapoptosis signaling is considered to be an antiviral pathway that is activated by the host to limit virus proliferation (40). To analyze the consequence of the apoptosis event induced by PRRSV, PAMs were exposed to PRRSV and synchronously treated with caspase3 inhibitor Z-DEVD-FMK. Compared with the control treatment, Z-DEVD-FMK effectively reduced the cleavage of caspase3, and, correspondingly, the level of PRRSV N was elevated at various hpi (Fig. 7B and C). These results tend to favor the previous conclusion that apoptosis induced by PRRSV is a host defense strategy by which to limit viral replication (41). To address whether the inhibition of apoptotic signals can relieve the restricted effect of MARCO on PRRSV, PAMs with MARCO overexpression were infected with PRRSV in the presence of caspase3 inhibitor Z-DEVD-FMK. As shown in Fig. 7D, the Z-DEVD-FMK treatment significantly inhibited MARCO-induced caspase3 cleavage and rescued the reduced PRRSV N expression, which suggests that MARCO inhibits virus infection through regulating the apoptotic signal induced by PRRSV. Taken together, these results demonstrate that the interaction between MARCO and GP5 may contribute to the PRRSV-induced apoptosis by which MARCO restrains PRRSV infections.

## DISCUSSION

Viral entry, consisting of the attachment to and the penetration into host target cells, is the first requisite for infection and is a critical event that governs the emergence of viruses in host populations. The intervention to disrupt the interactions between virus particle surface glycoproteins and particular cell receptors has become an extremely attractive therapy for viral infection. Here, the macrophage receptor with collagenous structure (MARCO), which is a member of the scavenger receptor family, was identified as one of the interactors of PRRSV GP5. MARCO is restricted to macrophages and primarily performs phagocytosis, which is an ancient and efficient defense mechanism for organisms to remove invaders (42). MARCO was originally identified by its ability to recognize and take up the oxidized low-density lipoprotein. However, it is now appreciated for its large repertoire of ligands. Ligands for MARCO include environmental particles, nucleic acids, bacterial lipopolysaccharides, viruses, oxidized lipoproteins, modified endogenous proteins, tumor cells, and dead cells or debris (43–47). These ligands often share the characteristic of being polyanionic, although macrophage-mediated phagocytosis lacks high specificity (22). The bacterial pathogens that are internalized by MARCO, such as Mycobacterium tuberculosis, Streptococcus pneumoniae, and Clostridium sordellii, are delivered to the lysosome to be digested (45, 48, 49). In the case of the virus, MARCO-mediated phagocytosis may lead to two different outcomes. The first is that the clearance of the virus from the circulation before the virus enters cells can impede viral dissemination. For example, depending on the binding of MARCO to the viral E2 glycoprotein, liver Kupffer cells phagocytize chikungunya virus in the mouse bloodstream, thereby relieving viremia (47). However, when encountering the permissive cells, the virus may coopt MARCO to expedite entry. The bindings of MARCO to HSV, vaccinia virus, and adenoviruses are examples in the latter category (24–26).

PAM is the primary cellular target for PRRSV, and it is the only porcine cell that is currently known to support PRRSV propagation *in vitro*. In this study, we found that MARCO was expressed in PAMs but not in Marc-145 cells, which further highlights the macrophage specificity characteristic of MARCO. Although the result that MARCO interacts with PRRSV GP5 was confirmed by pairwise Y2H testing, confocal microscopy, and co-IP, MARCO did not participate in the entry process of PRRSV into PAMs because neither blocking MARCO with a ligand nor overexpressing it directly impaired viral adsorption and internalization (Fig. 3). Furthermore, the significant colocalization of MARCO with the clathrin heavy chains in PAMs that were incubated with a ligand refuted the hypothesis that MARCO and PRRSV may enter into PAMs through different routes. Collectively, our obtained data demonstrate that MARCO may not serve as a potential receptor to mediate PRRSV entry.

Aside from the phagocytosis receptor, the emerging roles of MARCO in a variety of biological settings, such as macrophage polarization, inflammation response, antigen presentation, and cell apoptosis, have been identified (36, 50–53). Moreover, a previous study that analyzed PAM transcriptome differences revealed that MARCO expression is 2.48-fold higher in Tongcheng pigs than in large white pigs. Tongcheng pigs, a Chinese local breed, are known to have stronger resistance or tolerance to PRRSV than do large white pigs (54). Considering these, we explored the effect of MARCO on PRRSV, irrespective of the viral initial entry process. As expected, we found that MARCO was a host antiviral factor for PRRSV in PAMs. MARCO knockdown potentiated PRRSV infection, whereas its overexpression restricted virus infection in PAMs (Fig. 4). The results presented here further demonstrate the results of the above literature (54).

Apoptosis is a common programmed cell death. As is often the case with other viruses, PRRSV has been reported to induce PAMs apoptosis. We reconfirmed the phenomenon in this study. The apoptosis executioner protein caspase3 was activated from 12 hpi in PAMs, and at 24 hpi, 27.6% of PAMs were apoptotic (Fig. 6A and B). These suggest that PRRSV-induced apoptosis happens at the mid and later stages of infection. Next, we found that although MARCO itself could not trigger an apoptotic signal in PAMs, MARCO acted as a proapoptotic factor in PRRSV-infected PAMs. The knockdown of MARCO weakened, whereas overexpression exacerbated, PRRSV-induced apoptosis in PAMs (Fig. 6C–E). Similar to our results, the role of MARCO in apoptosis signaling has been ascertained in silica-induced pulmonary fibrosis, immune-modifying nanoparticle-exposed monocytes, and tumor-associated macrophages (37, 38, 55).

Previous reports showed that the GP5 of PRRSV is an inducer of apoptosis (8, 11, 56), and MARCO has been demonstrated to interact with GP5 and exacerbate apoptosis in PAMs. These remind us to investigate whether MARCO contributes to GP5-triggered apoptotic signal. In this study, we found that GP5 expression in Marc-145 cells caused 7.99% apoptosis cells, and the coexpression of GP5 and MARCO further elevated the percentage to 12.41% (Fig. 7A). These results confirm the previous conclusion and demonstrate that MARCO promotes GP5-induced apoptosis in Marc-145 cells. Due to the difficulty of GP5 expression in PAMs, we were unable to simulate PRRSV infection using GP5 expression to investigate the role of the interaction between MARCO and GP5 in apoptosis. We will further investigate this. Taking these results together, we conclude that the interaction between MARCO and PRRSV GP5 may contribute to the proapoptotic effect of MARCO.

In general, cell death following apoptosis has been identified as a powerful defense mechanism by which to curtail viral spread (57). However, in some cases, viruses exploit apoptosis to promote their release from infected cells. Thus, apoptosis is involved in the spread of virions from one infected cell to the next. In this study, we confirmed that apoptosis induced by PRRSV was deleterious for its propagation (Fig. 7B and C). Moreover, we discovered that reducing the cleavage level of caspase3 through Z-DEVD-FMK inhibition restored PRRSV N expression in PAMs with MARCO overexpression (Fig. 7D). This suggests that the regulation of apoptosis by MARCO is a mechanism by which MARCO restricts PRRSV infections in PAMs.

As an ancient pattern recognition receptor, MARCO is thought to be highly conserved within mammals (58). The MARCO proteins of human, mouse, and green monkey species indeed share a highly similar amino acid (aa) sequence. However, when these species were compared to pigs, the similarities of the MARCO aa sequences are only 53.2%, 48.7%, and 52.0%, respectively (data not shown). Moreover, the MARCO of pigs has lost about 100 aa in the collagenous domain. Hence, pig MARCO owns a shorter collagenous domain, compared with the MARCO of other species. It is due to the high variation of MARCO in pigs that there are currently no available anti-MARCO antibodies for our study. In this study, the intracellular domain of pig MARCO was identified as being important to its restrictive function on PRRSV. Interestingly, the MARCO lacking an intracellular region predominantly stayed in the cytoplasm and was no longer diffused on the plasma membrane, as the full-length MARCO was (Fig. 5). Remarkably, the previous study suggests that in both humans and mice, the SRCR domain of MARCO (accurately, the arginine residues in this domain) are associated with its transport to the cell surface (32). Unexpectedly, a pig MARCO mutant that was devoid of a SRCR domain could not be expressed in our study. Thus, here we are unable to determine whether the SRCR of pig MARCO is involved in its location on the cell surface. Together, these inconsistencies again uncover the specificity of pig MARCO. Importantly, the phenomenon that the intracellular domain of MARCO affects its subcellular localization resembles the TIM-1 protein, which is an authentic Dengue virus entry receptor, and this may drive our study further (59).

**FIG 8:**
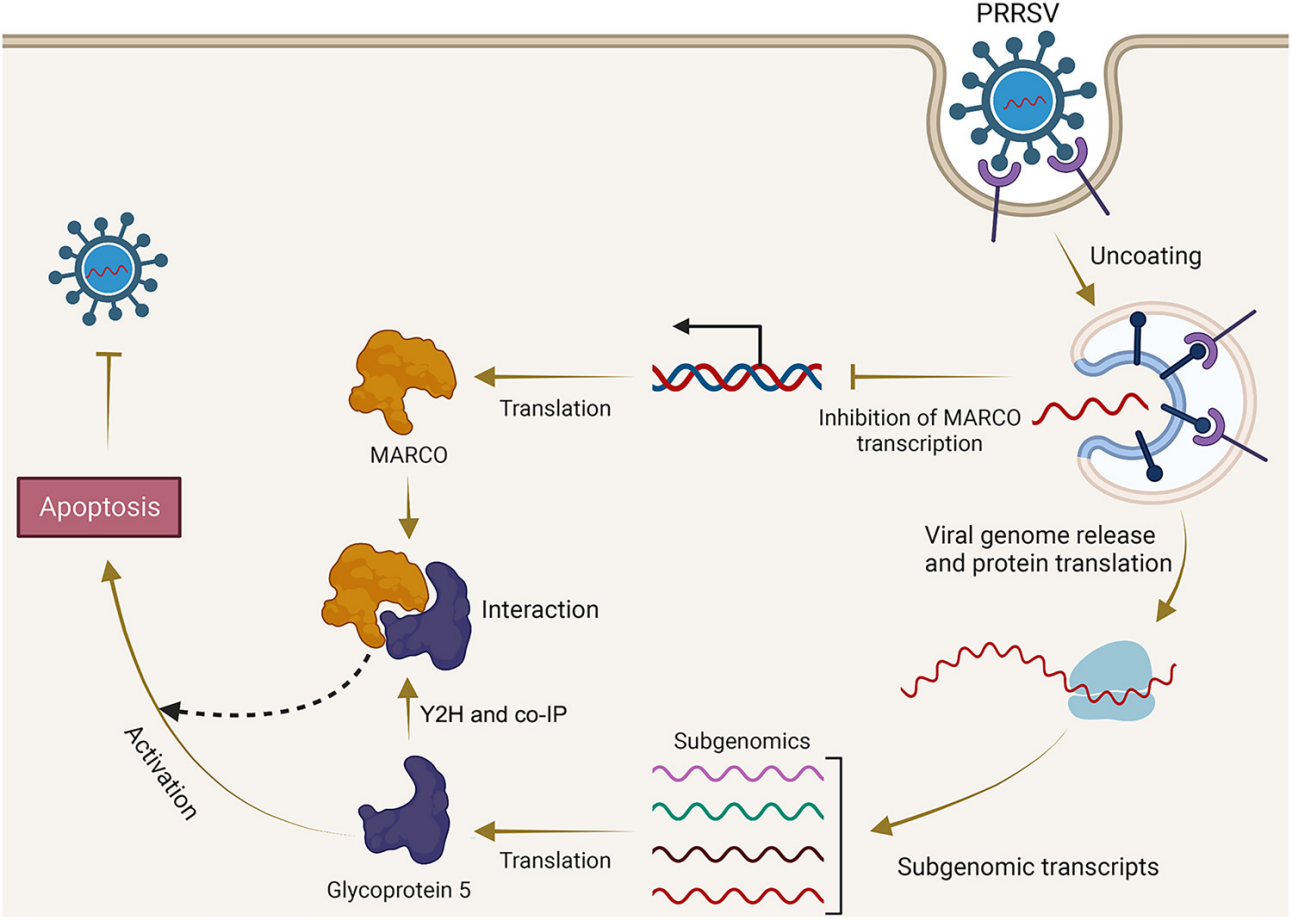
The schematic model of MARCO regulation of PRRSV infection. PRRSV infection downregulates the expression of MARCO, which acts as a host restriction factor for PRRSV. MARCO interacts with PRRSV GP5 and aggravates GP5-induced apoptosis. The apoptotic cells are not conducive to PRRSV propagation. Thus, MARCO inhibits PRRSV infection through the regulation of apoptosis.

In summary, our data reveal the role of MARCO during PRRSV infection (Fig. 8). MARCO is constitutively expressed in PAMs and is downregulated upon PRRSV infection. MARCO interacts with PRRSV GP5, which may contribute to GP5-induced apoptosis and thereby inhibit virus replication. Our work demonstrates for the first time that MARCO is an efficient antiviral factor during PRRSV infection. These findings should contribute to the understanding of the host defense mechanism against PRRSV.

## MATERIALS AND METHODS

### Ethics statement.

This research was approved by the Institutional Animal Care and Use Committee of Sun Yat-sen University. All of the animal experiments were performed according to the Guide for the Laboratory Animal Environment and Facilities (GB14925-2010/XG1-2011, National Laboratory Animal Standardization Technical Committee) to ensure the humane and ethical treatment of the animals.

### Cells and viruses.

Porcine alveolar macrophages (PAMs) were isolated from the bronchoalveolar lavage fluid of 4- to 6-week-old, PRRSV-negative piglets, as described previously (60). PAMs were cultured in RPMI 1640 medium (Thermo Fisher, 61870036) supplemented with 10% fetal bovine serum (FBS; Gibco, 160099133), 100 U/mL penicillin, and 100 mg/mL streptomycin. African green monkey kidney cells (Marc-145) and human embryonic kidney cells (HEK293T) were cultured in Dulbecco’s modified Eagle’s medium (DMEM; Corning, 10-013-CVRC) supplemented with 10% FBS. All of the cells were cultured at 37°C in an incubator with 5% CO_2_. The highly pathogenic PRRSV strain Li11 (GenBank accession number EF112445) was available in our laboratory. The PRRSV was propagated and titrated on Marc-145 cells.

### cDNA library construction and yeast two-hybrid screening.

To generate a porcine cDNA library for Y2H screening, the lung tissues from piglets that were challenged with or without PRRSV were collected and mixed. Total RNA was extracted from the pooled samples using the TRIzol reagent. After isolating the mRNA, the double-stranded cDNA was synthesized and cloned into each of the three reading frames of the pDONR222 plasmid through the gateway BP reaction, using a CloneMiner II cDNA Library Construction Kit (Thermo Fisher, A11180). Competent Escherichia coli DH10B was electrotransformed with the ligation products. Subsequently, the transformants were cultured in SOC medium. The transformed cells were harvested to serve as the entry cDNA library. Then, the recombination of the entry cDNA library with the pPR3-N-DEST vector was performed through the gateway LR reaction. DH10B was electrotransformed with these recombinant plasmids to finally obtain the cDNA library.

A DUALmembrane Y2H screening was carried out to identify the host proteins that interact with PRRSV-GP5, using the above porcine cDNA library. In brief, viral GP5 was cloned into a pBT3-SUC vector to generate the bait plasmid GP5-pBT3-SUC. The cDNA library was cloned into the pPR3-N-DEST vector to generate the prey plasmids. All of the cDNA library plasmids were transformed into the yeast strain Y2HGold, together with the GP5-pBT3-SUC plasmid, using a yeast transformation system. Then, the transformants were selected for growth on a synthetic defined (SD) medium lacking His, Leu, Trp, and Ade (SD/−4). Positive colonies were processed for sequencing to identify each bait-prey pair.

For every bait-prey pair identified by sequencing, pairwise Y2H testing was performed to ensure high reproducibility. The bait plasmid GP5-pBT3-SUC and prey plasmids were cotransformed into the yeast strain NMY51. The blue positive colonies that were grown on SD/−4 plates supplemented with X-gal indicated interaction pairs. In pairwise Y2H testing, the pair of pTSU2-APP and pNubG-Fe65 was used as a positive control, and the combination of the vector pPR3-N with GP5-pBT3-SUC or pTSU2-APP was included as a negative control.

### Plasmid transfection and adenovirus infection.

The expression plasmid for MARCO or PRRSV GP5 was constructed using the method of cohesive end ligation. In brief, coding sequences (CDS) of pig MARCO or PRRSV GP5 were amplified via PCR and then digested by a pair of appropriate restriction endonucleases. Meanwhile, the vector pcDNA3.1(+) M-Myc-C, pCMV-Myc-C, or pmCherry-N1 was digested with the same restriction endonucleases to produce the same cohesive end. Then, either MARCO or GP5 sequence was inserted into the vector with T4 ligase. The transfection of either MARCO or the GP5 recombinant plasmid into Marc-145 or 293T cells was performed using Lipofectamine 3000 (Thermo Fisher, L3000015), according to the manufacturer’s instructions. The primer sequences of the PCR are listed in Table S1.

To overexpress MARCO in PAMs, MARCO recombinant adenovirus was established using the AdMAX system. The CDS of MARCO was inserted into the shuttle plasmid pDC316-EGFP. Then, 293A cells were cotransfected with pDC316-EGFP-MARCO and the genomic plasmid pBHGlox(delta)E1,3Cre using Lipofectamine 3000. At 48 h posttransfection, a cytopathic effect (CPE) appeared. The culture medium was changed every 2 to 3 days until the CPE was completed. The 293A were harvested, centrifuged, and resuspended in HBSS buffer. Then, the cell suspension was frozen and thawed three times from −80°C to 37°C to completely release the adenovirus. Finally, the virus suspension was marked as P0 and stocked at −80°C. To propagate the MARCO recombinant adenovirus, 293A cells that were plated on a T175 culture flask were incubated with P0 recombinant adenovirus for 2 to 3 days until absolute CPE. The cells and culture supernatant were harvested together and centrifuged at 210 g/min, at 4°C for 15 min. The pellet was resuspended in the HBSS buffer. The virus titer was determined to be 10^10^ CFU/mL.

### siRNA knockdown.

The small interfering RNAs (siRNAs) against MARCO were synthesized by GenePharma (Shanghai, China). PAMs grown in a 6-well plate were transfected with MARCO-specific siRNAs and the corresponding control siRNA, using the Lipofectamine RNAiMAX transfection reagent (Thermo Fisher, 13778150). The final concentration of siRNA that was transfected into the cells was 50 nmol/L. At 24 h posttransfection, the cells were harvested to examine the knockdown efficiency via RT-qPCR analysis. The siRNA sequences are listed in Table S2.

### Viral titers.

The viral titers in the cell supernatants were assessed via TCID_50_ (50% tissue culture infective dose per mL). Briefly, the monolayers of Marc-145 cells seeded in 96-well plates were incubated with a series of 10-fold-diluted supernatant samples for 72 to 96 h. Then, CPE was recorded. TCID_50_ was calculated via the method of Reed-Muench.

### Reverse transcription-quantitative PCR (RT-qPCR).

Total RNA was extracted from cells using the TRIzol reagent (Magen, R4801). As for tissue, the samples were grounded in liquid nitrogen before TRIzol extraction. Then, 1 μg of the total RNA was reverse transcribed into cDNA using a GoScript RT Reagent Kit (Promega, A5001), according to the manufacturer’s instructions. Real-time PCR was conducted with SYBR green qPCR Mix (GenStar, A301) on a LightCycler 480 PCR system (Roche). The quantitation of all target gene expression was normalized to the endogenous reference gene HPRT in PAMs or to GAPDH in Marc-145 cells via the 2^−ΔCt^ method. The primer sequences that were used are listed in Table S1.

### Western blotting.

The cells were washed twice using cold PBS and were then lysed in Western lysis buffer (Beyotime, P0013) containing 1 mM proteinase inhibitor phenylmethanesulfonyl fluoride (PMSF; Beyotime, ST506). The cell lysates were denatured in 5 × SDS-PAGE sample loading buffer (Beyotime, P0015) at 95°C for 10 min, and the proteins were separated via SDS-PAGE electrophoresis. Then, the proteins were transferred to polyvinylidene difluoride membranes (PVDF; Millipore, IPVH00010) via a semidry transfer apparatus. Subsequently, membranes were blocked in blocking buffer (Beyotime, P0252) for 1 h at ambient temperature, and they were then incubated with primary antibody overnight at 4°C. The next day, after three washes in Tris-buffered saline and 0.1% Tween 20 (TBST), the membranes were incubated with the corresponding horseradish peroxidase-conjugated secondary antibodies for 1 h at ambient temperature. Following another three washes, the protein bands were visualized using an Enhanced Chemiluminescence (ECL) Kit (Fdbio Science, FD8000). Antibodies used in the Western blotting: anti-Myc antibody (1:2000; Cell Signaling Technology, 2276s), anti-PRRSV-N antibody (4A5) (1:3000; Jeno Biotech, 9041), anti-cleaved caspase3 antibody (1:1000; Cell Signaling Technology, 9664S), anti-PRRSV GP5 antibody (1:1000; GeneTex, GTX129062), anti-GAPDH antibody (1:3000; GeneTex, GTX100118), HRP-linked anti-mouse IgG (1:2000; Cell Signaling Technology, 7076), and HRP-linked anti-rabbit IgG (1:2000; Cell Signaling Technology, 7074).

### Immunofluorescence and confocal microscopy.

PAMs or Marc-145 cells that were grown in 12-well cell plates or in 15 mm covered, glass-bottomed petri dishes were washed with PBS and were fixed with 4% paraformaldehyde (Beyotime, P0099) for 10 min, and they were then permeabilized with 0.5% Triton X-100 (Beyotime, P0096) in PBS for 5 min. The cells were blocked in Immunol staining blocking buffer (Beyotime, P0260) for 30 min at room temperature. Subsequently, the primary antibodies were incubated overnight at 4°C. After washing to remove unbound antibodies, the cells were incubated with the appropriate Alexa Fluor 488 or 555 conjugated secondary antibodies at room temperature for 1 h in the dark. Washing three times again, the cell nuclei were stained with 4′,6-diamidino-2-phenylindole (DAPI; Solarbio, C0065) for 5 min. Finally, the fluorescence images were taken using an inverted fluorescence microscope (NIKON ECLIPSE Ti2-U) or a confocal laser scanning microscope (TCS-SP5, LEICA). Antibodies used in the immunofluorescence and confocal microscopy: anti-Myc antibody (1:800; Cell Signaling Technology, 2276s), anti-PRRSV N antibody (4A5) (1:1500; Jeno Biotech, 9041), anti-clathrin heavy chain (1:1000, Abcam, ab21679), anti-Mouse IgG Alexa Fluor 488 (1:1000, Cell Signaling Technology, 4408s), and anti-Rabbit IgG Alexa Fluor 555 (1:1000, Cell Signaling Technology, 4413s).

### Coimmunoprecipitation.

293T cells cotransfected with plasmids expressing Myc-tagged MARCO and mCherry-tagged PRRSV-GP5 were washed twice with cold PBS and lysed in 300 μL of the IP lysis buffer (Beyotime, P0013) supplemented with 1 mM PMSF on ice for 20 min. Then, the lysates were centrifuged at 12,000 × *g* at 4°C for 15 min. 30 μL of the lysate supernatant was reserved as the whole lysate sample and denatured in the SDS-PAGE sample loading buffer at 95°C for 10 min. The immunoprecipitate was prepared, according to the instructions from the Dynabeads Protein G Immunoprecipitation Kit (Thermo Fisher, 10007D). Briefly, the remaining lysate supernatant was incubated with anti-Myc antibody or isotype control normal IgG at 4°C for 3 h, and this was followed by incubation with magnetic beads at room temperature for 1 h. After washing the antigen-antibody-magnetic beads complex three times, the combined proteins were eluted from the beads with 20 μL elution buffer at 95°C. Finally, the immunoprecipitate, together with the whole lysate proteins, were subjected to Western blotting, as described above.

### Flow cytometry analysis of apoptosis.

Apoptosis was examined using a FITC Annexin V Apoptosis Detection Kit (BD, 556547), according to the manufacturer’s instructions. Briefly, 1 × 10^6^ PAMs or Marc-145 cells were digested with 0.25% Tyrisin without EDTA and washed twice with cold PBS. The cells were resuspended in 1 mL of 1× binding buffer, and 100 μL of the cell solution was transferred to a 1.5 mL EP tube. Then, 5 μL of Annexin V-FITC and 5 μL of propidium iodide (PI) were added per tube. The cells were incubated for 15 min at room temperature in the dark. After incubation, 300 μL of 1× binding buffer were supplemented to each tube. A flow cytometric analysis was performed immediately after staining. The data were acquired and analyzed using CytoFLEX S (Beckman).

### Statistical analysis.

The data were presented as means ± standard errors (SE). The statistical analyses were performed via two-tailed unpaired *t* tests, using the GraphPad Prism software package. *P* values are indicated by asterisks in the figures as follows: *, *P < *0.05; **, *P < *0.01; and ***, *P < *0.001. All of the cellular experiments were repeated at least three times.

## References

[1] An T-Q, Li J-N, Su C-M, Yoo D. 2020. Molecular and cellular mechanisms for PRRSV pathogenesis and host response to infection. Virus Res 286:197980. doi:10.1016/j.virusres.2020.197980. 32311386 PMC7165118

[2] Lunney JK, Fang Y, Ladinig A, Chen N, Li Y, Rowland B, Renukaradhya GJ. 2016. Porcine reproductive and respiratory syndrome virus (PRRSV): pathogenesis and interaction with the immune system. Annu Rev Anim Biosci 4:129–154. doi:10.1146/annurev-animal-022114-111025. 26646630

[3] Kappes MA, Faaberg KS. 2015. PRRSV structure, replication and recombination: origin of phenotype and genotype diversity. Virology 479-480:475–486. doi:10.1016/j.virol.2015.02.012. 25759097 PMC7111637

[4] Fang Y, Snijder EJ. 2010. The PRRSV replicase: exploring the multifunctionality of an intriguing set of nonstructural proteins. Virus Res 154:61–76. doi:10.1016/j.virusres.2010.07.030. 20696193 PMC7114499

[5] Huang C, Zhang Q, Feng W-h. 2015. Regulation and evasion of antiviral immune responses by porcine reproductive and respiratory syndrome virus. Virus Res 202:101–111. doi:10.1016/j.virusres.2014.12.014. 25529442 PMC7132515

[6] Dokland T. 2010. The structural biology of PRRSV. Virus Res 154:86–97. doi:10.1016/j.virusres.2010.07.029. 20692304 PMC7114433

[7] Popescu LN, Trible BR, Chen N, Rowland RRR. 2017. GP5 of porcine reproductive and respiratory syndrome virus (PRRSV) as a target for homologous and broadly neutralizing antibodies. Vet Microbiol 209:90–96. doi:10.1016/j.vetmic.2017.04.016. 28528961

[8] Suárez P, Díaz-Guerra M, Prieto C, Esteban M, Castro JM, Nieto A, Ortín J. 1996. Open reading frame 5 of porcine reproductive and respiratory syndrome virus as a cause of virus-induced apoptosis. J Virol 70:2876–2882. doi:10.1128/JVI.70.5.2876-2882.1996. 8627762 PMC190145

[9] Gagnon CA, Lachapelle G, Langelier Y, Massie B, Dea S. 2003. Adenoviral-expressed GP5 of porcine respiratory and reproductive syndrome virus differs in its cellular maturation from the authentic viral protein but maintains known biological functions. Arch Virol 148:951–972. doi:10.1007/s00705-002-0943-y. 12721802 PMC7087108

[10] Wissink EHJ, Kroese MV, van Wijk HAR, Rijsewijk FAM, Meulenberg JJM, Rottier PJM. 2005. Envelope protein requirements for the assembly of infectious virions of porcine reproductive and respiratory syndrome virus. J Virol 79:12495–12506. doi:10.1128/JVI.79.19.12495-12506.2005. 16160177 PMC1211556

[11] Music N, Gagnon CA. 2010. The role of porcine reproductive and respiratory syndrome (PRRS) virus structural and non-structural proteins in virus pathogenesis. Anim Health Res Rev 11:135–163. doi:10.1017/S1466252310000034. 20388230

[12] Duan X, Nauwynck HJ, Pensaert MB. 1997. Effects of origin and state of differentiation and activation of monocytes/macrophages on their susceptibility to porcine reproductive and respiratory syndrome virus (PRRSV). Arch Virol 142:2483–2497. doi:10.1007/s007050050256. 9672608 PMC7086874

[13] Calvert JG, Slade DE, Shields SL, Jolie R, Mannan RM, Ankenbauer RG, Welch S-KW. 2007. CD163 expression confers susceptibility to porcine reproductive and respiratory syndrome viruses. J Virol 81:7371–7379. doi:10.1128/JVI.00513-07. 17494075 PMC1933360

[14] Kim HS, Kwang J, Yoon IJ, Joo HS, Frey ML. 1993. Enhanced replication of porcine reproductive and respiratory syndrome (PRRS) virus in a homogeneous subpopulation of MA-104 cell line. Arch Virol 133:477–483. doi:10.1007/BF01313785. 8257302

[15] Vázquez-Calvo A, Saiz J-C, McCullough KC, Sobrino F, Martín-Acebes MA. 2012. Acid-dependent viral entry. Virus Res 167:125–137. doi:10.1016/j.virusres.2012.05.024. 22683298

[16] Delputte PL, Costers S, Nauwynck HJ. 2005. Analysis of porcine reproductive and respiratory syndrome virus attachment and internalization: distinctive roles for heparan sulphate and sialoadhesin. J Gen Virol 86:1441–1445. doi:10.1099/vir.0.80675-0. 15831956

[17] Delputte PL, Vanderheijden N, Nauwynck HJ, Pensaert MB. 2002. Involvement of the matrix protein in attachment of porcine reproductive and respiratory syndrome virus to a heparinlike receptor on porcine alveolar macrophages. J Virol 76:4312–4320. doi:10.1128/jvi.76.9.4312-4320.2002. 11932397 PMC155060

[18] Van Gorp H, Van Breedam W, Delputte PL, Nauwynck HJ. 2008. Sialoadhesin and CD163 join forces during entry of the porcine reproductive and respiratory syndrome virus. J Gen Virol 89:2943–2953. doi:10.1099/vir.0.2008/005009-0. 19008379

[19] Zhang Q, Yoo D. 2015. PRRS virus receptors and their role for pathogenesis. Vet Microbiol 177:229–241. doi:10.1016/j.vetmic.2015.04.002. 25912022

[20] Kraal G, van der Laan LJ, Elomaa O, Tryggvason K. 2000. The macrophage receptor MARCO. Microbes Infect 2:313–316. doi:10.1016/s1286-4579(00)00296-3. 10758408

[21] Arredouani MS. 2014. Is the scavenger receptor MARCO a new immune checkpoint? Oncoimmunology 3:e955709. doi:10.4161/21624011.2014.955709. 25941575 PMC4292731

[22] Canton J, Neculai D, Grinstein S. 2013. Scavenger receptors in homeostasis and immunity. Nat Rev Immunol 13:621–634. doi:10.1038/nri3515. 23928573

[23] Murphy JE, Tedbury PR, Homer-Vanniasinkam S, Walker JH, Ponnambalam S. 2005. Biochemistry and cell biology of mammalian scavenger receptors. Atherosclerosis 182:1–15. doi:10.1016/j.atherosclerosis.2005.03.036. 15904923

[24] MacLeod DT, Nakatsuji T, Yamasaki K, Kobzik L, Gallo RL. 2013. HSV-1 exploits the innate immune scavenger receptor MARCO to enhance epithelial adsorption and infection. Nat Commun 4:1963. doi:10.1038/ncomms2963. 23739639 PMC3681428

[25] Maler MD, Nielsen PJ, Stichling N, Cohen I, Ruzsics Z, Wood C, Engelhard P, Suomalainen M, Gyory I, Huber M, Müller-Quernheim J, Schamel WWA, Gordon S, Jakob T, Martin SF, Jahnen-Dechent W, Greber UF, Freudenberg MA, Fejer G. 2017. Key role of the scavenger receptor MARCO in mediating adenovirus infection and subsequent innate responses of macrophages. mBio 8:e00670-17. doi:10.1128/mBio.00670-17. 28765216 PMC5539421

[26] MacLeod DT, Nakatsuji T, Wang Z, di Nardo A, Gallo RL. 2015. Vaccinia virus binds to the scavenger receptor MARCO on the surface of keratinocytes. J Invest Dermatol 135:142–150. doi:10.1038/jid.2014.330. 25089661 PMC4268046

[27] Fraser I, Hughes D, Gordon S. 1993. Divalent cation-independent macrophage adhesion inhibited by monoclonal antibody to murine scavenger receptor. Nature 364:343–346. doi:10.1038/364343a0. 8332192

[28] Arredouani MS, Franco F, Imrich A, Fedulov A, Lu X, Perkins D, Soininen R, Tryggvason K, Shapiro SD, Kobzik L. 2007. Scavenger receptors SR-AI/II and MARCO limit pulmonary dendritic cell migration and allergic airway inflammation. J Immunol 178:5912–5920. doi:10.4049/jimmunol.178.9.5912. 17442975

[29] Nicoletti A, Caligiuri G, Törnberg I, Kodama T, Stemme S, Hansson GK. 1999. The macrophage scavenger receptor type A directs modified proteins to antigen presentation. Eur J Immunol 29:512–521. doi:10.1002/(SICI)1521-4141(199902)29:02<512::AID-IMMU512>3.0.CO;2-Y. 10064066

[30] Ye N, Wang B, Feng W, Tang D, Zeng Z. 2022. PRRS virus receptors and an alternative pathway for viral invasion. Virus Res 320:198885. doi:10.1016/j.virusres.2022.198885. 35948131

[31] Sankala M, Brännström A, Schulthess T, Bergmann U, Morgunova E, Engel J, Tryggvason K, Pikkarainen T. 2002. Characterization of recombinant soluble macrophage scavenger receptor MARCO. J Biol Chem 277:33378–33385. doi:10.1074/jbc.M204494200. 12097327

[32] Brännström A, Sankala M, Tryggvason K, Pikkarainen T. 2002. Arginine residues in domain V have a central role for bacteria-binding activity of macrophage scavenger receptor MARCO. Biochem Biophys Res Commun 290:1462–1469. doi:10.1006/bbrc.2002.6378. 11820786

[33] Cholewa J, Nikolic D, Post SR. 2010. Regulation of Class A scavenger receptor-mediated cell adhesion and surface localization by PI3K: identification of a regulatory cytoplasmic motif. J Leukoc Biol 87:443–449. doi:10.1189/jlb.0509318. 19952357 PMC2830124

[34] Bowdish DM, Gordon S. 2009. Conserved domains of the class A scavenger receptors: evolution and function. Immunol Rev 227:19–31. doi:10.1111/j.1600-065X.2008.00728.x. 19120472

[35] Costers S, Lefebvre DJ, Delputte PL, Nauwynck HJ. 2008. Porcine reproductive and respiratory syndrome virus modulates apoptosis during replication in alveolar macrophages. Arch Virol 153:1453–1465. doi:10.1007/s00705-008-0135-5. 18563285

[36] Thakur SA, Hamilton RF, Holian A. 2008. Role of scavenger receptor A family in lung inflammation from exposure to environmental particles. J Immunotoxicol 5:151–157. doi:10.1080/15476910802085863. 18569385

[37] Eisinger S, Sarhan D, Boura VF, Ibarlucea-Benitez I, Tyystjärvi S, Oliynyk G, Arsenian-Henriksson M, Lane D, Wikström SL, Kiessling R, Virgilio T, Gonzalez SF, Kaczynska D, Kanatani S, Daskalaki E, Wheelock CE, Sedimbi S, Chambers BJ, Ravetch JV, Karlsson MCI. 2020. Targeting a scavenger receptor on tumor-associated macrophages activates tumor cell killing by natural killer cells. Proc Natl Acad Sci USA 117:32005–32016. doi:10.1073/pnas.2015343117. 33229588 PMC7750482

[38] Yang M, Wang N, Li W, Li H, Zhao Y, Yao S, Chen W. 2019. Therapeutic effects of scavenger receptor MARCO ligand on silica-induced pulmonary fibrosis in rats. Toxicol Lett 311:1–10. doi:10.1016/j.toxlet.2019.04.026. 31028789

[39] Hamilton RF, Thakur SA, Mayfair JK, Holian A. Jr., 2006. MARCO mediates silica uptake and toxicity in alveolar macrophages from C57BL/6 mice. J Biol Chem 281:34218–34226. doi:10.1074/jbc.M605229200. 16984918

[40] Danthi P. 2016. Viruses and the diversity of cell death. Annu Rev Virol 3:533–553. doi:10.1146/annurev-virology-110615-042435. 27501259

[41] Huo Y, Fan L, Yin S, Dong Y, Guo X, Yang H, Hu H. 2013. Involvement of unfolded protein response, p53 and Akt in modulation of porcine reproductive and respiratory syndrome virus-mediated JNK activation. Virology 444:233–240. doi:10.1016/j.virol.2013.06.015. 23850458

[42] Medzhitov R, Janeway CA. Jr. 1997. Innate immunity: impact on the adaptive immune response. Curr Opin Immunol 9:4–9. doi:10.1016/s0952-7915(97)80152-5. 9039775

[43] Arredouani MS, Palecanda A, Koziel H, Huang Y-C, Imrich A, Sulahian TH, Ning YY, Yang Z, Pikkarainen T, Sankala M, Vargas SO, Takeya M, Tryggvason K, Kobzik L. 2005. MARCO is the major binding receptor for unopsonized particles and bacteria on human alveolar macrophages. J Immunol 175:6058–6064. doi:10.4049/jimmunol.175.9.6058. 16237101

[44] Dahl M, Bauer AK, Arredouani M, Soininen R, Tryggvason K, Kleeberger SR, Kobzik L. 2007. Protection against inhaled oxidants through scavenging of oxidized lipids by macrophage receptors MARCO and SR-AI/II. J Clin Invest 117:757–764. doi:10.1172/JCI29968. 17332894 PMC1804372

[45] Bowdish DME, Sakamoto K, Kim M-J, Kroos M, Mukhopadhyay S, Leifer CA, Tryggvason K, Gordon S, Russell DG. 2009. MARCO, TLR2, and CD14 are required for macrophage cytokine responses to mycobacterial trehalose dimycolate and Mycobacterium tuberculosis. PLoS Pathog 5:e1000474. doi:10.1371/journal.ppat.1000474. 19521507 PMC2688075

[46] Xing Q, Feng Y, Sun H, Yang S, Sun T, Guo X, Ji F, Wu B, Zhou D. 2021. Scavenger receptor MARCO contributes to macrophage phagocytosis and clearance of tumor cells. Exp Cell Res 408:112862. doi:10.1016/j.yexcr.2021.112862. 34626585

[47] Carpentier KS, Davenport BJ, Haist KC, McCarthy MK, May NA, Robison A, Ruckert C, Ebel GD, Morrison TE. 2019. Discrete viral E2 lysine residues and scavenger receptor MARCO are required for clearance of circulating alphaviruses. Elife 8:e49163. doi:10.7554/eLife.49163. 31596239 PMC6839921

[48] Dorrington MG, Roche AM, Chauvin SE, Tu Z, Mossman KL, Weiser JN, Bowdish DME. 2013. MARCO is required for TLR2- and Nod2-mediated responses to Streptococcus pneumoniae and clearance of pneumococcal colonization in the murine nasopharynx. J Immunol 190:250–258. doi:10.4049/jimmunol.1202113. 23197261 PMC3529821

[49] Thelen T, Hao Y, Medeiros AI, Curtis JL, Serezani CH, Kobzik L, Harris LH, Aronoff DM. 2010. The class A scavenger receptor, macrophage receptor with collagenous structure, is the major phagocytic receptor for Clostridium sordellii expressed by human decidual macrophages. J Immunol 185:4328–4335. doi:10.4049/jimmunol.1000989. 20810988 PMC7682803

[50] Zhang L, Nie L, Cai S-Y, Chen J, Chen J. 2018. Role of a macrophage receptor with collagenous structure (MARCO) in regulating monocyte/macrophage functions in ayu, Plecoglossus altivelis. Fish Shellfish Immunol 74:141–151. doi:10.1016/j.fsi.2017.12.059. 29305330

[51] Braun BJ, Slowik A, Leib SL, Lucius R, Varoga D, Wruck CJ, Jansen S, Podschun R, Pufe T, Brandenburg L-O. 2011. The formyl peptide receptor like-1 and scavenger receptor MARCO are involved in glial cell activation in bacterial meningitis. J Neuroinflammation 8:11. doi:10.1186/1742-2094-8-11. 21299846 PMC3040686

[52] Georgoudaki A-M, Prokopec KE, Boura VF, Hellqvist E, Sohn S, Östling J, Dahan R, Harris RA, Rantalainen M, Klevebring D, Sund M, Brage SE, Fuxe J, Rolny C, Li F, Ravetch JV, Karlsson MCI. 2016. Reprogramming tumor-associated macrophages by antibody targeting inhibits cancer progression and metastasis. Cell Rep 15:2000–2011. doi:10.1016/j.celrep.2016.04.084. 27210762

[53] Granucci F, Petralia F, Urbano M, Citterio S, Di Tota F, Santambrogio L, Ricciardi-Castagnoli P. 2003. The scavenger receptor MARCO mediates cytoskeleton rearrangements in dendritic cells and microglia. Blood 102:2940–2947. doi:10.1182/blood-2002-12-3651. 12842997

[54] Liang W, Ji L, Zhang Y, Zhen Y, Zhang Q, Xu X, Liu B. 2017. Transcriptome differences in porcine alveolar macrophages from Tongcheng and large white pigs in response to highly pathogenic porcine reproductive and respiratory syndrome virus (PRRSV) infection. Int J Mol Sci 18:1475. doi:10.3390/ijms18071475. 28704922 PMC5535966

[55] Lai C, Chadban SJ, Loh YW, Kwan TK-T, Wang C, Singer J, Niewold P, Ling Z, Spiteri A, Getts D, King NJC, Wu H. 2022. Targeting inflammatory monocytes by immune-modifying nanoparticles prevents acute kidney allograft rejection. Kidney Int 102:1090–1102. doi:10.1016/j.kint.2022.06.024. 35850291

[56] Dea S, Gagnon CA, Mardassi H, Pirzadeh B, Rogan D. 2000. Current knowledge on the structural proteins of porcine reproductive and respiratory syndrome (PRRS) virus: comparison of the North American and European isolates. Arch Virol 145:659–688. doi:10.1007/s007050050662. 10893147 PMC7087215

[57] Kvansakul M. 2017. Viral infection and apoptosis. Viruses 9:356. doi:10.3390/v9120356. 29168732 PMC5744131

[58] Yap NVL, Whelan FJ, Bowdish DME, Golding GB. 2015. The evolution of the scavenger receptor cysteine-rich domain of the class A scavenger receptors. Front Immunol 6:342. doi:10.3389/fimmu.2015.00342. 26217337 PMC4491621

[59] Dejarnac O, Hafirassou ML, Chazal M, Versapuech M, Gaillard J, Perera-Lecoin M, Umana-Diaz C, Bonnet-Madin L, Carnec X, Tinevez J-Y, Delaugerre C, Schwartz O, Roingeard P, Jouvenet N, Berlioz-Torrent C, Meertens L, Amara A. 2018. TIM-1 ubiquitination mediates dengue virus entry. Cell Rep 23:1779–1793. doi:10.1016/j.celrep.2018.04.013. 29742433

[60] Zhu Z, Zhang X, Dong W, Wang X, He S, Zhang H, Wang X, Wei R, Chen Y, Liu X, Guo C. 2020. TREM2 suppresses the proinflammatory response to facilitate PRRSV infection via PI3K/NF-κB signaling. PLoS Pathog 16:e1008543. doi:10.1371/journal.ppat.1008543. 32401783 PMC7250469

