# Supplementary material for: MARCO Inhibits Porcine Reproductive and Respiratory Syndrome Virus Infection through Intensifying Viral GP5-Induced Apoptosis
Source: Microbiol Spectr. 2023 Apr 20;11(3):e04753-22. doi: 10.1128/spectrum.04753-22 (PMC10269733; doi:10.1128/spectrum.04753-22)
Supplement: Supplemental file 1 — Tables S1 and S2. Download spectrum.04753-22-s0001.pdf, PDF file, 0.1 MB [file spectrum.04753-22-s0001.pdf]

S1 Table. Primers used in this study

Primer name	Genbank number	Sequence of primer (5'- 3')
**Primers for PCR**		
MARCO-Myc-F	NM_001256366.1	CCCAAGCTTGGGATGTTGCCTAAAGTGGAGACCTTT
MARCO-Myc-R		CCGGAATTCGCTGCAGTTCACGCCTGCGTC
MARCO-(27-395) -Myc-F	NM_001256366.1	CCCAAGCTTGGGATGTGGTTCATGTTCCTGGTGGT
MARCO-(27-395) -Myc-R		CCGGAATTCGCTGCAGTTCACGCCTGCGTC
Mcherry-GP5-F	EF112445	CTCGAGGCCACCATGTTGGAGAAATGCTT
Mcherry-GP5-R		GGATCCCGAGGACGACCCCATTGTTCCG
GP5-Myc-F	EF112445	CTCGAGGCCACCATGTTGGAGAAATGCTT
GP5-Myc-R		GGATCCCGAGGACGACCCCATTGTTCCG
**Primers for qRT-PCR**		
PRRSV-N-F	EF112445	AAAACCAGTCCAGAGGCAAG
PRRSV-N-R		CGGATCAGACGCACAGTATG
PHPRT-F	XM_021079504.1	TGGAAAGAATGTCTTGATTGTTGAAG
PHPRT-R		ATCTTTGGATTATGCTGCTTGACC
GAPDH-F	XM_007967342.1	TGACAACAGCCTCAAGATCG
GAPDH-R		GTCTTCTGGGTGGCAGTGAT
MARCO-F	NM_001256366.1	GCCAAAGGATCTTCTGGGCT
MARCO-R		CAGAAGACAGTGGCATCCGA

S2 Table. Sequences of siRNA in this study

siRNA	Direction	Sequence (5'to 3')
siMARCO-1	Sense	GGUGAUGACAGUUCUGAAATT
	Antisense	UUUCAGAACUGUCAUCACCTT
siMARCO-2	Sense	CCUCUUUCCUCCAGAGCAUTT
	Antisense	AUGCUCUGGAGGAAAGAGGTT
siMARCO-3	Sense	GCAUGUAAAGGGAGCGAAUTT
	Antisense	AUUCGCUCCCUUUACAUGCTT
